# Legume Lectins: Proteins with Diverse Applications

**DOI:** 10.3390/ijms18061242

**Published:** 2017-06-12

**Authors:** Irlanda Lagarda-Diaz, Ana Maria Guzman-Partida, Luz Vazquez-Moreno

**Affiliations:** 1 CONACyT-Universidad de Sonora, Blvd. Luis Encinas y Rosales, Hermosillo, Sonora 83000, Mexico; Irlanda.lagarda@unison.mx; 2 Coordinacion de Ciencia de los Alimentos, Centro de Investigacion en Alimentacion y Desarrollo, A.C., Apartado Postal 1735, Hermosillo, Sonora 83304, Mexico; gupa@ciad.mx

**Keywords:** lectin, legume, insecticidal, antimicrobial, cancer

## Abstract

Lectins are a diverse class of proteins distributed extensively in nature. Among these proteins; legume lectins display a variety of interesting features including antimicrobial; insecticidal and antitumor activities. Because lectins recognize and bind to specific glycoconjugates present on the surface of cells and intracellular structures; they can serve as potential target molecules for developing practical applications in the fields of food; agriculture; health and pharmaceutical research. This review presents the current knowledge of the main structural characteristics of legume lectins and the relationship of structure to the exhibited specificities; provides an overview of their particular antimicrobial; insecticidal and antitumor biological activities and describes possible applications based on the pattern of recognized glyco-targets.

## 1. Introduction

Currently, lectins are defined as carbohydrate binding proteins of non-immune origin that can recognize and bind simple or complex carbohydrates in a reversible and highly specific manner. For a long time, these proteins were named hemagglutinins, due to their ability to agglutinate red blood cells. At present, this concept is used when the specificity is unknown. It is also known that monomeric lectins do not exhibit this agglutinating activity [1]. Lectins are ubiquitously present in fungi, animals, bacteria, viruses and plants. Among plant lectins, those of legumes have been the most widely considered [2]. These lectins are abundant in the seeds and belong to a group of highly homologous proteins. However, their carbohydrate specificities and quaternary structures vary extensively [3], which greatly influences the type of recognized targets.

Throughout the history of lectin research, legume seeds have been screened for lectin activity. The existence of binding sites for specific carbohydrates, the main characteristic of lectins, is undoubtedly an important factor for determining their activity and future applications based on their properties. This review centers on the lectins of legumes, highlighting their specificity, particularities, main reported activities and potential applications.

## 2. Structure of Legume Lectins

**Figure 1:**
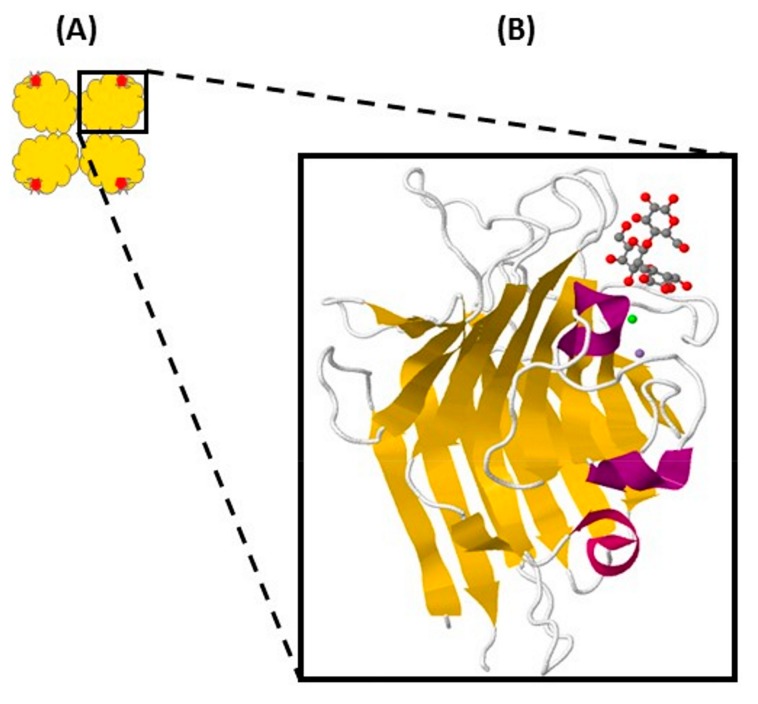
Overall structure of legume lectins. (**A**) tetramer with carbohydrate recognition domains (red); (**B**) amplified image of monomer with β-sheets (yellow), α-turns (purple), metal binding sites (area with green and gray spheres) and carbohydrate recognition domain (area occupied by the grey and red molecule). Image modified from Protein Data Bank (accession code 1GZ9).

Legume lectins represent the largest family of carbohydrate binding proteins, and their physicochemical and biological properties have been broadly studied [4,5,6,7,8]. Research of the plant lectin family using newer biochemical and biophysical strategies has significantly moved the field forward, and provided a model framework for studying protein–carbohydrate interactions. Although plant lectins share high sequence homology and exhibit comparable physicochemical and structural properties, the diversity of their carbohydrate recognition specificity is remarkable. Generally, the three-dimensional structure of plant lectins is characterized by β-sheets that are connected by α turns, β turns and bends. In addition to short loops, the quaternary interfaces are formed between β-sheets. The β-sheets are connected by loops forming antiparallel chains usually devoid of α helices (Figure 1) [4,5,9,10,11,12].

Analysis of legume lectin monomers reveals high similarity in sequence as well as structure, with only minor variations in the length of loops and strands. The monomer structures display a jellyroll motif best described as a sandwich of 25–30 kDa containing a carbohydrate recognition domain (CRD) and metal binding sites for divalent cations (Ca^2+^ and Mn^2+^). However, in spite of strong similarities in primary, secondary and tertiary structures, the quaternary structures show considerable variations that lead to changes in the monomer–monomer interactions and the presence/absence of protein modifications, such as glycosylation. The impact of these variations has functional implications as they dictate the specificity of multivalent glycan binding [1,5,11,13,14,15,16,17,18]. Most legume lectins appear to assemble as homodimers or homo-tetramers (dimers of dimers), the stability of which is attributed to hydrophobic cooperation, hydrogen bonds and salt links [4,9,19].

Legume lectins are subdivided into two categories, those with indistinguishable or almost indistinguishable subunits, and those with a variety of subunits [20]. *Phaseolus vulgaris* lectins (PHA) are well-studied lectins of the first group [21,22,23,24,25,26]. There are more than forty structures of legume lectins reported in either the apo (ligand-free) or holo (sugar-bound) form in the Protein Data Bank [15,26,27,28,29,30,31,32]. However, data about lectins from wild legumes is rare. 

Mexico has roughly 1800 species of wild legumes confined to various regions, and more than 33 percent are within the Sonoran Desert [5,33,34,35,36,37,38,39,40,41,42]. The isolation and purification of lectins from seeds of native plants such as *Parkinsonia aculeata*, *Olneya tesota*, *Acacia constricta*, *Prosopis juliflora*, *Cercidium praecox*, *Caesalpinia caladenia* and *Phaseolus acutifolius* has been described. Lectins from these plants have monomers of either 30 or 66 kDa that also can form tetramers or dimers. In addition, it has been shown that these lectins involve a group of different isolectins, compared to those found in lectins of the *Phaseolus* genus [43,44,45,46,47].

On the other hand, the primary sequences of plant lectins have been used to infer evolutionary relationships, to reflect processing routes, to suggest folding patterns as well as to provide clues on how carbohydrate ligands fit into their binding pockets [20]. Direct amino acid sequencing has revealed homology among the lectins from the wild desert legumes *A. constricta* [6] and *O. tesota* [5,48], and more domesticated species like *P. vulgaris* and *P. coccineus* [49,50]. Furthermore, antibodies raised against PHA show reactivity with lectins from *A. constricta*, *C. praecox* and *C. caladenia* [6]. From these studies, it is clear that lectins have been conserved during the evolution of legumes and that the extensive homologies reflect taxonomical relationships among these plants [51].

## 3. Specificity of Legume Lectins

Legume lectins represent a crucial point in the study of the molecular basis of protein–carbohydrate interactions. Each has a CRD with a basic architecture accessible to both monosaccharides and/or oligosaccharides that confers remarkable divergence in their specificities [12,52]. Lectins can non-covalently interact with carbohydrates in a manner that is usually reversible and highly specific. Recognition can occur in the terminal or intermediate position of the glycan structure [53]. In all legume lectins, four invariant amino acid residues participate in the binding of the carbohydrate, yet each lectin shows different specificity [18,54,55]. The CRD pocket is formed by four loops adjacent to each other, but the loops are not close in sequence. These regions exhibit the highest residue variability and appear to be involved in specificity determination [56,57]. The CRD of legume lectins lies in close proximity to the metal binding sites, which may aid in binding activity [18], but are not often directly involved in carbohydrate binding [18].

Most lectins bind to mono- and oligosaccharides; however, more recently discovered lectins show specificity for complex sugars and glycoproteins. Generally, lectins show specificity for di-, tri- and tetra-saccharides with association constants significantly higher than those for the corresponding monosaccharides [5,18,58]. Some lectins have shown affinity towards carbohydrate structures not present in plants, such as the Thomsen-nouveau antigen or complex N-glycan structures with terminal galactose and sialic acid residues [30]. The specificity of legume lectins for some typical animal glycans, has led to the suggestion that legume lectins play a role in plant defense against insects and/or predator animals [59,60].

The legume lectins that have been purified and characterized as members of the complex type include, *Griffonia simplicifolia* IV lectin (GS IV) [5,61], *Maackia amurensis* lectin (MAH and MAL-1) [62], *Cicer arietinum* lectin [63], *Saraca indica* lectin [58,64] and *P. vulgaris* E and L lectins (PHA-E and PHA-L) [5,23,26,65,66,67,68,69]. Wild legume lectins from the Sonoran Desert also have shown varying degrees of specificity for some complex carbohydrates of glycoproteins. The hemagglutinating activity of *A. constricta* isolectins (VLs) and the *P. juliflora* lectin is strongly inhibited in the presence of asialofetuin and to a lesser extent with fetuin and thyroglobulin [6,40]. *C. praecox* and *C. caladenia* lectins are inhibited by fetuin and mucine [37]. *O. tesota* PF1 and PF3 lectins are inhibited by free monosaccharides and/or by complex carbohydrates of fetuin, thyroglobulin, immunoglobulin, mucine and ovalbumin, whereas PF2 is only inhibited by the intact glycoprotein [5]. Although glycan structures present in glycoproteins are known to have N and O linked oligosaccharides, desialylation of the glycan can result in an increase or decrease in lectin binding affinity [70]. The galactose residues found in glycosylated proteins are often capped with sialic acid, a characteristic that may alter the interaction with lectins, as is seen for *Amaranthus caudatus* and *Abrus pulchellus* [71,72].

Many studies on lectin–carbohydrate specificity have been published. Unfortunately, often specificity analyses are used to “specifically” determine structural motifs in glycoconjugates without taking into account the rather complex data on this subject. Furthermore, the choice of ligands used to assay lectin binding in some studies is limited either by natural or commercially available sources of glycans, or to simple mono- or disaccharides. Therefore, a critical re-evaluation of lectin binding specificities is required. Such studies using microarray techniques with immobilized glycans or immobilized lectins, dot blot assays to screen different lectins and antibodies with multiple oligosaccharides, combined with information gleaned using recombinant glycosyltransferases, are opening up new possibilities of generating a broader range of standard (neo)glycoconjugates with clearly defined structures [73].

## 4. Antimicrobial Activity

Antimicrobials have been used to treat diseases caused by bacteria and fungi and such treatments have significantly reduced the mortality rate from these infections in humans and animals. However, the extensive use of antimicrobial substances in medical, agricultural, and veterinary practices is a topic of great concern to clinical microbiologists all over the world due to the growing emergence of opportunistic microorganism strains resistant to drugs that cause serious infections [74,75,76,77]. Microbial resistance is a genetic phenomenon resulting in a reduction in effectiveness of drug therapy; it may be caused by mutations during the reproductive process of the microorganisms or by imported genes acquired through transduction, conjugation, and transformation mechanisms [78]. Therefore, there is a growing interest in developing new strategies for inhibiting the growth or survival of microorganisms based on the screening of new sources of natural compounds as alternatives to commercially available drugs [74,79].

A large number of proteins with antibacterial, antifungal and/or antiviral activity have been isolated from the seeds, tubers, and rhizomes of different plants, where they accumulate to high levels and may function as a reserve source of amino acids and metal ions [80]. These proteins may directly interfere with the growth, multiplication and spread of microbial agents by different mechanisms [81], as in the case of lectins, by agglutination and/or microorganism immobilization. Plant lectins often are present at potential sites of microbial invasion and their binding to fungal structures led to the inhibition of fungal growth and germination. Studies carried out with soy bean and common lentil agglutinins provide evidence for these roles [51,82,83,84]. A main characteristic of lectins is their ability to interact with glyco-components present on the cell membrane surface, in cytoplasmic and nuclear structures and in the extracellular matrix of cells and tissues from almost all kingdoms of life [85]. Therefore, plant lectins are thought to play important roles in the plant immune defense and emerge as potential antimicrobial candidates for drug therapies. The antimicrobial effect of legume lectins is shown in Table 1. Although many lectins show antimicrobial activity, clinical trials are needed to establish therapeutic efficacy, to optimize dosage, delivery and bioavailability, and to assess potential allergic reactions [86].

### 4.1. Bacteria

The antibacterial activity of lectins results from their interaction with a wide variety of complex carbohydrates of the bacterial cell wall, such as teichoic and teichuronic acids, peptidoglycans and lipopolysaccharides [2,85]. Lectins of *Triticum vulgare*, *Dolichos lablab* L., *Trigonella foenumgraecum*, *Trifolium alexandrium* L., *Bauhinia variegata* L. and *Delonix regia* have shown antimicrobial activity against *Mycobacterium rhodochrous*, *Bacillus cereus*, *B. megaterium*, *B. sphaericus*, *Escherichia coli*, *Serratia marcescens*, *Corynebacterium xerosis* and *Staphylococcus aureus* [86] Lectins from the *Vicieae tribe* strongly react with the bacterial cell wall components, muramic acid, *N*-acetylmuramic acid and muramyl dipeptides [87]. Nevertheless, although legume seed lectins can recognize and bind to the constituents of the bacterial cell wall, such binding does not imply that these interactions occur in vivo, and certainly does not prove that these lectins are involved in the protection of seedlings against bacteria [51].

It is thought that plant lectins do not alter the membrane structure and/or permeability nor disturb the normal intracellular processes of invading microbes, since they exert an indirect effect that is based on interactions with cell wall carbohydrates or extracellular glycans [60]. However, electron microscopy studies revealed that treatment with *Araucaria angustifolia* lectin promoted morphologic alterations, including the presence of pores in the membrane of Gram-positive bacteria, and bubbling on the cell wall of Gram-negative bacteria [88]. Others reported that the antibacterial activity of lectins occurred through the interactions of the lectins with *N*-acetylglucosamine, *N*-acetylmuramic acid and tetrapeptides linked to *N*-acetylmuramic acid present in the cell wall of Gram-positive bacteria, or to lipopolysaccharides present in the cell wall of Gram-negative bacteria [87].

In addition to these findings, lectins from the legumes from *Canavalia ensiformis*, *T. foenumgraecum*, *Arachis hypogaea*, *Cajanus cajan*, *P. vulgaris* and *Pisum sativum* were shown to inhibit biofilm formation by *Streptococcus mutans*, but the growth of planktonic cells was not affected [89]. Since bacterial biofilms make disinfection procedures more difficult by increasing bacterial resistance to detergents and antibiotics, the inhibition of biofilm formation and development by legume lectins could be a useful characteristic of these proteins. Furthermore, the rapid evolution of bacteria with antibiotic resistance (in particular, multidrug resistant bacteria or superbugs) has reduced the efficacy of conventional treatments against their biofilms. Hence, development of non-antibiotic alternatives that could efficiently treat or reduce biofilms should become a priority. Furthermore, research focused on finding lectins that reduce superbugs could represent an interesting alternative drug therapy that deserves further attention.

### 4.2. Fungi and Yeasts

Regardless of the considerable number of lectins that have been characterized, only a small number have shown antifungal effects. Because plant lectins do not penetrate the cell wall or the membrane to reach the cytoplasm of fungi, direct inhibition of fungal growth by lectins is unlikely. In spite of this, indirect responses produced by the attachment of lectins to chitin and other glycans on the fungal surface could affect fungal survival or other activities [105,106].

Lectin binding to hyphae could result in inhibition of fungi growth as a result of poor nutrient absorption as well as by interference with the spore germination process [107]. Lectins can cause different morphological changes that render fungi more vulnerable to differing stress conditions [108]. For example, in one study, lectin interactions resulted in swollen hyphae, vacuolization of the cell content and improved lysis of hyphal cell wall, that, in turn, increased susceptibility of fungi to osmotic shock [107]. In another case, it was suggested that small antifungal lectins could penetrate the fungal cell wall and reach the cell membrane where blocking of active sites of enzymes could alter cell wall morphogenesis [108,109].

Some reports indicate that lectins from the legumes *Astragalus mongholicus*, *P. coccineus*, *Archidendron jiringa* Nielsen, *B. ungulata*, *Glycine max*, *Indigofera heterantha* and *A. hypogaea* can exhibit antifungal activity against various species of phytopathogenic fungi, such as *Botrytis cinerea*, *Fusarium oxysporum*, *F. moniliforme*, *F. solani*, *Colletorichum* sp., *Drechslera turcia*, and *Exserohilum turcicum*, as well as pathogenic fungi such as *Candida albicans*, *Penicillium italicummm*, and *Aspergillus* sp. [82,91,92,93,110,111]. Additional studies are required to elucidate the molecular basis for the antifungal activity of individual lectins as the ability of lectins to inhibit growth differs among fungal species.

### 4.3. Virus

Plant lectins with antiviral activity are of substantial therapeutic interest. Some of these carbohydrate binding proteins exhibit significant activity against human immunodeficiency virus (HIV) and other viruses [97]. Retroviruses, such as HIV, have a surface covered by highly glycosylated virally-encoded glycoproteins, e.g., gp120 (that contains high mannose and/or hybrid glycans) and gp41. The carbohydrates on these proteins are particularly important because the glycans can be used as tools to render the virus easily recognized by the immune system, and thus susceptible to immunological neutralization [112]. The interactions between host and proteins from the viral envelop also can be altered by compounds that specifically recognize and bind glycans. Furthermore, lectins can crosslink surface viral glycans and thereby prevent interactions with other co-receptors [83].

Antiviral lectins used as therapeutic agents offer lower toxicity and can be included in topical applications. Generally, lectins are odorless, and resistant to high temperatures and low pH, ideal properties for development of microbicide drugs [86].

Most current antiviral therapeutics prevent the viral life cycle, or inhibit the entrance of the virus into host cells [113]. The antiviral action of plant lectins varies extensively depending upon their carbohydrate specificity. Corona virus are highly susceptible to mannose specific lectins that interfere with the viral attachment in early phases of the replication cycle and suppress viral development by binding towards the end of the viral infection cycle [114]. Other antiviral non-legume lectins with encouraging properties include griffithsin (GRFT), cyanovirin (CV-N) and banana lectin (BanLec), and BanLec has been recommended as an antiviral microbicide. Most often antiviral lectins are recommended for incorporation into vaginal and rectal gels, creams or suppositories that act as a barrier to prevent HIV transmission. In such processes, lectins bind the virus preventing its entry and fusion to target cells, consequently averting contamination [115].

Several legume lectins possess antiviral activity. Lectins like *C. ensiformis* agglutinin (Con A), *Lens culinaris* agglutinin (LCA), *Vicia faba* agglutinin, *P. sativum* agglutinin (PSA) and PHA-E bind to the envelope glycoprotein gp120 and to inhibit fusion of HIV-infected cells with CD4 cells by a carbohydrate-specific interaction with the HIV-infected cells [103]. In addition, *G. max* agglutinins inhibit HIV-1 reverse transcriptase activity [116]. Although glycan structures of viral proteins involve high mannose, further research in this area is needed to explore lectins that recognize structures with different sugars such as sialic acid, fucose and *N*-acetyl glucosamine with the aim of developing novel approaches and therapies in the field of virus biology. 

## 5. Insecticidal Activity

Legume lectins are toxic to a broad spectrum of insects representing several orders including, Coleoptera, Diptera, Lepidoptera, Hymenoptera, Isoptera, Neuroptera and Homoptera. In this regard, the family of leguminous lectins is the most studied due to its insecticidal potential. A great number of legume lectins have shown insecticidal activity including those from *C. ensiformis*, *P. sativum*, *P. vulgaris*, *Glechoma hederacea*, *G. simplicifolia*, *O. tesota* (PF2) and *B. monandra* [8,117,118].

These lectins showed harmful effects in the different developmental stages of insects (larvae and adults). In this regard, they may increase mortality, delay development and/or adult emergence and reduce fecundity. Usually, the effect of lectins on insects is evaluated by feeding larvae with artificial seeds containing the lectin or with transgenic plants that overexpress the lectin of interest. The expression of a given lectin in transgenic plants is only possible if the lectin molecular sequence is well characterized and also requires an available transformation protocol for the desired plant species [119].

**Figure 2:**
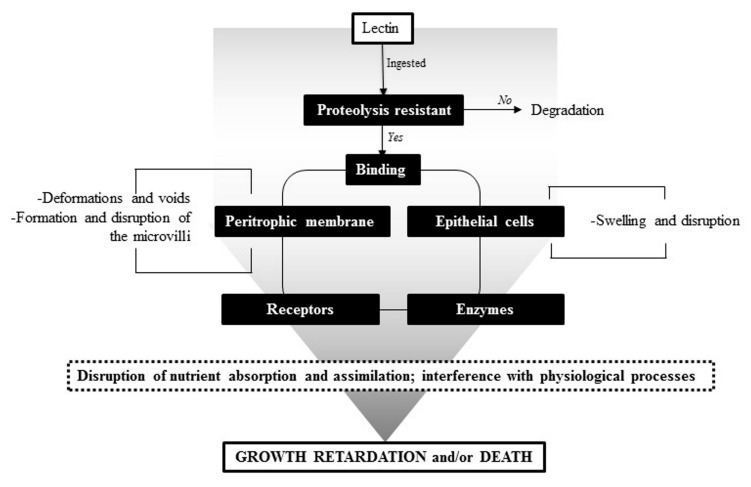
Insecticidal action mechanism for lectins.

In the last twenty years, research has focused on elucidating the mode of action of insecticidal lectins. In general, lectins can exert a toxic effect via binding to the peritrophic membrane (PM), peritrophic gel (PG) or the brush-border microvilli of epithelial cells (Figure 2) [120]. The PM or PG is a film surrounding the food bolus in most insects and is composed of chitin and proteins. Some non-legume lectins with insecticidal activity such as the *Rhizoctonia solani* agglutinin and *Sambucus nigra* agglutinin II can pass through the PM of the red flour beetle, *Tribolium castaneum*. This ability to reach the endoperitrophic space is governed by the dimensions of the molecule and the charge and size of the PM pores [121]. 

In the case of insects lacking a PM, the insecticidal effect of lectins may be exerted by their direct interaction with glycoconjugates present on the cell epithelium [122]. In fact, the insecticidal activity of lectins relies on their binding properties to sugars present on the surface of the insect gut epithelial cells. In insects, the surface of epithelial cells is rich in glycoproteins that, in addition to their determinant roles for the proper gut function, provide a number of available targets for lectin binding.

The interaction of lectins with different glycoproteins or glycan structures in insects may interfere with important physiological processes (Table 2). Although the key receptors for lectins could be localized on the surface of gut epithelial cells, if lectins are internalized, they may interact with a new set of targets located in the intracellular space, allowing the lectin to interfere with particular metabolic pathways. Such internalization has been more studied for non-legume lectins. For example, the garlic leaf lectin, which affects survival and development of the moth *Helicoverpa armigera*, was found to be internalized by binding to a glycosylated alkaline phosphatase anchored to the insect midgut membrane. In addition, *Galantus nivalis* lectin was reported to cross the midgut epithelium of *Nilaparvata lugens* and co-localize in the fat body and hemolymph. Such co-localization was proposed to occur via a carrier-receptor, ferritin. The internalization of the *Colocasia esculenta* tuber agglutinin into insect hemolymph also was demonstrated, suggesting it could interact with various *N*-glycosylated intracellular targets, which may, in part, explain the lectin toxicity [122]. Although the complete mechanism by which some lectins are able to cross midgut epithelial cells is not yet fully understood, available evidence has highlighted the implication of clathrin-mediated endocytosis as part of this process [123,124].

Regardless of the mechanisms involved in lectin interactions with insect cells, in order to be toxic, a lectin must avoid degradation by the digestive enzymes from the insect gut. The insecticidal effect of some lectins is attributed to the resistance of these proteins to proteolysis and to their properties of stability in a wide pH range. For example, PF2, an insecticidal lectin to *Zabrotes subfasciatus*, is resistant to in vitro proteolysis by insect digestive enzymes. This is in contrast to PHA-E lectin that is digested after 4 h of incubation with these enzymes, and therefore is not toxic for this insect [8]. Other lectins with insecticidal activity that share the common feature of resistance to proteolysis by different digestive enzymes include, *G. simplicifolia II* lectin (GS II)*, Ulex europeus* agglutinin (UEA) and *B. monandra* lectin [117,118]. The degree of resistance to digestive enzymes depends on the capacity of the lectin to bind to glycoconjugates of the insect gut. GS II mutant lectins lacking the ability to bind carbohydrates displayed sensitivity to proteolysis by digestive enzymes with a consequent loss of toxicity [125].

In summary, the insecticidal effect exhibited by several plant lectins is a complex phenomenon that relies on the capacity of the lectin to interfere with critical physiological processes during insect development. Such interference often may be mediated by a lack of nutrient absorption that occurs when midgut epithelial cells are atrophied as a result of the interaction of lectins with glyco-targets located either on the surface or inside the cell. Studies of the recognition of plant lectins by insect gut glyco-targets can allow one to develop hypotheses on which cellular pathways are affected by the insecticidal activity. These pathways vary depending on the type of insect and the lectin, and could be as diverse as the type of recognized glycan or receptors, and might include the effectors of energy metabolism, and redox and ionic homeostasis (summarized in Table 2).

Finally, the study of potential non-yet-identified insecticidal plant lectins may contribute to the development of distinctive tools for sustainable pest control. However, in this regard, care must be taken considering the effect lectins might exert on beneficial insects as well.

## 6. Antitumor Activity

Recently, an increased interest in the potential of plant lectins as cancer therapeutic agents has become evident. Lectins can be used to study the metastatic distribution patterns, the expression profiles of cancer cells glycoconjugates and their biological effects in various tumors. It is well known that carbohydrates present on the cell membrane are important for cell recognition, communication and adhesion. In cancer, these interactions are essential for tumor progression and metastasis. In tumor cells, glycosylation is generally altered in comparison with normal cells and these alterations can be detected by lectins [135,136]. Litynska et al. compared the lectin-binding pattern in different human melanoma cell lines using the PHA-L lectin and other non-legume lectins, including the *Galanthus nivalis* lectin, *S. nigra* lectin, MAL, *Datura stramonium* agglutinin and wheat germ agglutinin (WGA). Studies demonstrated that acquisition of metastatic potential of tumor cells is correlated with the expression of branched and sialylated complex *N*-oligosaccharides [137]. Interestingly, the binding of MAL-I to gastric cancer cells was proportional to their metastatic capacity, and a high expression of α (2,3)-linked sialic acids was closely correlated with lymph node metastasis [137]. Korourian et al. used *G. simplicifolia* lectin-I (GS I) and *Vicia vilosa* agglutinin (VVA) to establish an expression analysis of carbohydrate antigens in human breast ductal carcinoma in situ (DCIS). Both lectins showed a significant association with nuclear grade (cell size and uniformity) of DCIS [138]. Positive correlations also were found between the expression of VVA- and GSI-reactive structures and tumor grade in DCIS patients, suggesting that the glycoconjugates found in these antigens are associated with invasiveness in DCIS [138]. PHA can be used to discriminate between hepatocellular carcinoma and benign liver disease [139]. This suggests that lectins could potentially be used to develop prognostic tools for cancer diagnosis and the detection of metastasis, and to some extent to estimate the severity of the clinical case. 

In addition to these uses, plant lectins have shown in vivo and in vitro antitumor activity. Lectins with the potential to induce inhibition of tumor cell growth, such as, Con A, Mistletoe type-I lectins and PHA are currently in different phases of clinical trials [138,140]. Plant lectins can modify the expression of interleukins and some protein kinases and in this way modulate the immune system. Furthermore, in cancers, lectins could alter the signaling pathways involved in the expression of members of the Bcl-2, Autophagy (ATG) related, and caspase families, as well as p53, ERK, Ras-Raf, and BNIP3, and thereby induce both apoptosis and autophagy [141]. Soybean lectin produces reactive oxygen species in a dose-dependent manner in HeLa cells inducing apoptosis, autophagy and DNA damage in the cells [142].

The cell surface carbohydrates are key targets for lectins; in cancer, a general pathway involves recognition of carbohydrate receptors triggering the activation of enzymes such as MBL-associated serine proteases [143]. However, the binding of lectins to glycans on the cell membrane may not be sufficient to induce apoptosis. Kim et al. showed that cell death requires lectin endocytosis that triggers signaling for apoptosis [143]. Con A is cytotoxic to hepatoma cell lines. Its toxicity is mediated primarily by its binding to the mannose moiety of cell membrane glycoproteins, with subsequent internalization and accumulation in the mitochondria. Autophagy is then activated, leading to lysosomal degradation of the affected mitochondria and, ultimately, cell death [144,145]. Overall, these findings of cancer research have revealed the mechanisms responsible for the antitumor action of different plant lectins. These mechanisms are varied and depend upon different factors such as the tumor cell origin and type as well as lectin concentration.

## 7. Conclusions

The screening and characterization of novel lectins from legume resources has allowed the discovery of proteins with a great capacity to distinguish specific glycans from a diversity of glyco-targets found in a variety of organisms. Legume lectins could be potential candidates for developing tools based on protein–carbohydrate interactions with variable specificities. Lectin-mediated drugs focused on targeting specific cells could lead to promising anticancer and antimicrobial treatments, which would directly impact areas of economic importance, such as the pharmaceutical and food industries and agriculture. Additionally, the use of lectins against insect pests could provide a form of sustainable pest control.

More research is needed to explore and support the therapeutic effect of lectins. Studies of action mechanisms, the relationship between the role of the lectins and their molecular features, and finally, their effect in modulating the expression of proteins and genes are required to move forward the use of lectins for clinical applications.

Domesticated legumes provide an accessible and abundant source of lectins, unlike wild legumes, which, in some regions, may even be considered protected species. In some cases, successful recombinant production of lectins would be a key factor in whether a specific lectin could find use in a feasible industrial application. The overexpression of recombinant lectins remains a great challenge, even more so for those lectins where the lack of adequate post-translational modifications could compromise their activity.

## Figures and Tables

**Table 1:** Antimicrobial effect of legume lectins.

Lectin	Carbohydrate/Glycoprotein Receptor	Type of Microorganism	Mechanism of Action	Reference
From Leguminosae, tribe: *Vicieae* *Diocleae* *Phaseoleae* *Erithrinea* *Glycineae* *Sophoreae* *Galegeae* *Genisteae* *Loteae* *Acacieae*	Components of the cell Wall: Muramic acid, *N*-acetylmuramic acid, *N*-acetylglucosamine, Muramyl-dipeptides, glucosaminyl-muyamyl-dipeptide, lipopolysacharides	Bacteria	Form a channel or pore on cell membrane and the cell dies as a result of the out flowing of cellular contents. Bacterial aggregation and inhibition bacterial cell division	[79,83,87,89,90,91]
*Astragalus mongholicus* agglutinin *P. vulgaris agglutinin* *P. coccineus* agglutinin Soy bean agglutinin Peanut agglutinin	Components of fungal cell wall: Chitin, sialic acid	Fungi	Binding to hyphae, swollen hyphal, vacuolization of the cell content, and enhanced susceptibility to cell wall lysis of the hyphal induced by osmotic shock, producing more susceptibility to other stress conditions. This condition produces poor absorption of nutrients, interference spore germination and rupture of the cell wall.	[79,83,91,92,93,94,95,96,97,98]
Concanavalin A *Psophocarpus tetragonolubus* agglutinin *Lens culinaris* agglutinin *Vicia faba* agglutinin *Pisum sativum* agglutinin Erythroagglutinin	Components of viral envelope: Glycoproteins Gp120/Gp41, sialic acid	Virus	Bind to the glycosylated envelope protein and block cellular entry (interfere with replication cycle)	[98,99,100,101,102,103,104,105]

**Table 2:** Receptors of plant lectins of different insects.

Lectin	Receptor	Insect	Reference
*Allium sativum* L. bulbs (ASAI and ASAII)	Aminopeptidase N Sucrase	*Acyrthosiphon pisum*	[126]
*Galantus nivalis* lectin	Ferritin	*Nilaparvata lunges Spodoptera littoralis*	[127] [128]
*Myracrodruon urundeuva* leaf lectin (MuLL)	Trypsin α-amylase	*Aedes aegypti*	[129]
*Bauhinia monandra* leaf lectin (BmoLL)	α-amylase	*Callosobruchus maculatus*	[117]
Concanavalin A	β-glucosidases cathepsin L	*Rhopalosiphum padi L.*	[130]
*Allium sativum* Leaf Agglutinin (ASAL)	(Nicotinamide adenine dinucleotide reduced) quinone oxidoreductase	*Brown planthopper*	[131]
*Colocasia esculenta* tuber agglutinin (CEA)	Vacuolar ATP synthase ATP synthase Heat shock protein 70 clathrin heavy chain	*Lipaphis erysimi*	[122]
	Sarcoplasmic endoplasmic reticulum typ Ca^2+^ATPase	*Bemisia tabaci*	[122]
PF2 lectin	α-amylase V-type proton ATPase Arginine kinase Prohibitin Polyubiquitin Actin ATP-dependent RNA helicase ATP synthase subunit alpha Mitochondrial-processing peptidase α-tubulin Odorant receptor Cytochrome c oxidase	*Zabrotes subfasciatus*	[132] [133]
*Allium sativum* lectin	Aminopeptidase Cadherin-N Polycalin Alkaline phosphatase Cytochrome P450	*Helicoverpa* *Armigera*	[134]
	Alanyl Aminopeptidase N Sucrase	*Acyrthosiphon* *Pisum*	[134]

## Abbreviations

CRD	Carbohydrate recognition domain
PM	Peritrophic membrane
PG	Peritrophic gel
PHA	*Phaseolous vulgaris* agglutinin
MAL-1	*Maackia amurensis* lectin 1
DCIS	Human breast ductal carcinoma in situ
GS IV	*Griffonia simplicifolia* IV lectin
MAH and MAL-1	*Maackia amurensis* lectins
VLs	*Acacia constricta* isolectins
WGA	Wheat germ agglutinin
LCA	*Lens culinaris* agglutinin
PSA	*Pisum sativum* agglutinin
GS II	*Griffonia simplicifolia* II lectin
UEA	*Ulex europeus* agglutinin
ASAI and ASAII	*Allium sativum* L. bulbs
MuLL	*Myracrodruon urundeuva* leaf lectin
BmoLL	*Bauhinia monandra* leaf lectin
ASAL	*Allium sativum* Leaf Agglutinin
CEA	*Colocasia esculenta* tuber agglutinin
GS I	*Griffonia simplicifolia* lectin-I
VVA	*Vicia vilosa* agglutinin
